# Early-onset neonatal sepsis as a risk factor for peri-intraventricular hemorrhage in premature infants

**DOI:** 10.1590/1980-549720240013

**Published:** 2024-03-18

**Authors:** Mariana Martins Denicol, Vanessa Bielefeldt Leotti, Cátia Rejane Soares de Soares, Juliana Balbinot Hilgert

**Affiliations:** I Universidade Federal do Rio Grande do Sul, Postgraduate Program in Epidemiology – Porto Alegre (RS), Brazil.; II Grupo Hospitalar Conceição, Hospital da Criança Conceição – Porto Alegre (RS), Brazil.

**Keywords:** Premature infant, Neonatal sepsis, Neonatal intensive care units, Cerebral intraventricular hemorrhage, Prematuro, Sepse neonatal, Unidade de tratamento intensivo neonatal, Hemorragia cerebral intraventricular

## Abstract

**Objective:**

To assess early-onset sepsis as a risk factor of peri-intraventricular hemorrhage in premature infants born at less than or equal to 34 weeks’ gestation and admitted to a neonatal intensive care unit (NICU).

**Methods:**

This retrospective cohort study included premature patients born at less than or equal to 34 weeks’ gestation who were admitted to the NICU of a tertiary hospital in southern Brazil, and born from January 2017 to July 2021. Data were collected from patients’ medical records. Early-onset sepsis was measured according to the presence or absence of diagnosis within the first 72 hours of life, whereas the outcome, peri-intraventricular hemorrhage, was described as the presence or absence of hemorrhage, regardless of its grade.

**Results:**

Hazard ratios were calculated using Cox regression models. A total of 487 patients were included in the study, of which 169 (34.7%) had some degree of peri-intraventricular hemorrhage. Early-onset sepsis was present in 41.6% of the cases of peri-intraventricular hemorrhage, which revealed a significant association between these variables, with increased risk of the outcome in the presence of sepsis. In the final multivariate model, the hazard ratio for early-onset sepsis was 1.52 (95% confidence interval 1.01–2.27).

**Conclusion:**

Early-onset sepsis and the use of surfactants showed to increase the occurrence of the outcome in premature children born at less than or equal to 34 weeks’ gestation. Meanwhile, factors such as antenatal corticosteroids and gestational age closer to 34 weeks’ gestations were found to reduce the risk of peri-intraventricular hemorrhage.

## RESUMO

**Objetivo:**

O objetivo do presente trabalho foi avaliar a sepse precoce como fator de risco para hemorragia peri-intraventricular (HPIV) em prematuros com 34 semanas ou menos, admitidos em Unidade de Terapia Intensiva (UTI) Neonatal.

**Métodos:**

Este estudo de coorte retrospectivo incluiu pacientes prematuros com 34 semanas ou menos, que receberam alta da UTI Neonatal de hospital terciário, no sul do Brasil, nascidos no período de janeiro de 2017 a julho de 2021. Os dados foram coletados por meio dos prontuários desses pacientes. A sepse precoce foi mensurada conforme a presença ou a ausência do diagnóstico nas primeiras 72 horas de vida. Já o desfecho, hemorragia peri-intraventricular, foi descrito conforme a presença ou ausência da hemorragia, independentemente do grau.

**Resultados:**

*Hazard ratios* (HR) foram calculados por meio de modelos de regressão de Cox. Foram incluídos no estudo 487 pacientes. Destes, 169 (34,7%) apresentaram algum grau de hemorragia peri-intraventricular. A sepse precoce esteve presente em 41,6% dos casos de hemorragia peri-intraventricular e apresentou associação significativa, elevando o risco do desfecho quando presente. No modelo multivariável final, o HR para a sepse precoce foi de 1,52 (intervalo de confiança de 95% — IC95% 1,01–2,27).

**Conclusão:**

Sepse precoce e uso de surfactante demonstraram aumentar a ocorrência do desfecho em crianças prematuras até 34 semanas, enquanto fatores como corticoide antenatal e idades gestacionais mais próximas a 34 semanas mostraram reduzir o risco de ocorrência hemorragia peri-intraventricular.

## INTRODUCTION

Prematurity is a complex clinical syndrome and, as such, it should be addressed with multiple prevention strategies. It is a process that begins long before pregnancy, being determined by socioeconomic factors, work styles, and lifestyles, which interact complexly with biological factors that determine premature birth, according to Victora^1^. The World Health Organization defines as preterm infants those born before 37 weeks’ gestation. These newborns may be further classified as extremely premature, born at less than 28 weeks’ gestation; very premature, born from 28 to 32 weeks’ gestation; and moderate to late premature, from 32 to 37 weeks’ gestation^2^. There has been a strong trend of increase in preterm births in Brazil from the 1990s onwards^3^. It is estimated that 15 million babies are born premature worldwide every year. According to Perin et al., nearly 1 million children die every year due to premature birth complications^4^.

Neonatal sepsis remains a significant global problem, with few advances despite great efforts to solve it^5^. Prematurity, low birth weight, chorioamnionitis, premature prolonged rupture of membranes, resuscitation, low APGAR score, inability to breastfeed, prolonged hospital stay, and invasive procedures are among the risk factors^6^. Neonatal sepsis is divided into early-onset sepsis (EOS), when occurring within the first 24 to 72 hours of life, and late-onset sepsis (LOS), when occurring after the first 72 hours of life. EOS is mainly related to pre- and perinatal factors, among which gestational age stands out as an important predictor^7^. LOS, in turn, is related to post natal factors and multiple intensive care unit (ICU) procedures to which newborns are subjected, such as placement of catheters and endotracheal tubes, venous puncture, parenteral nutrition, congenital transmission, and transmission from the health care staff^8^.

Diagnosis of EOS is often difficult, because its signs and symptoms are inespecific^9^. The most common risk factors associated with EOS are birth before 37 weeks’ gestation, rupture of membranes for 18 hours or longer, group B streptococcus colonization in pregnant women not given intrapartum prophylactic antibiotictherapy, chorioamnionitis, maternal fever in the 48 hours preceding labor, cerclage or use of pessary, fetal medicine procedures in the 72 hours prior to child birth, and maternal urinary tract infection, either untreated or treated, for less than 72 hours^10^. The diagnosis of neonatal sepsis is based on a combination of clinical presentation, the use of non-specific markers, blood cultures, and the use of molecular methods, including C-protein reaction (PCR)^11^. The presence of germs in the cultures is considered gold standard for diagnosis; although positivity is low, the positive culture in blood or cerebrospinal fluid confirms the diagnosis^9^. Therefore, in several cases it is necessary to assume the diagnosis and implement the treatment based on clinical findings and laboratory examinations^9,11^.

Intracranial hemorrhage is an important problem in newborns, especially in premature infants and those with birth weight below 1,500g^12^. The pathogenesis of peri-intraventricular hemorrhage (PIVH) is complex and heterogeneous, being attributed to intrinsic fragility of germinal matrix vasculature and to fluctuation in the cerebral blood flow. The presence of associated coagulation disorders leads to an increased risk of hemorrhage. Situations such as vaginal birth, low Apgar score, severe ventilatory impairment, patent ductus arteriosus, and infections are associated with increased fluctuations in cerebral blood flow and thus represent important risk factors for the development of hemorrhage^13^. Extremely premature and extremely low birth weight infants are born at a critical time in brain maturation, and improving neurological development outcomes remains a challenge^14^. Tests to identify these changes should be indicated for all children at risk of developing injuries due to pre-, peri-, and postnatal causes^15^. Intracranial hemorrhage has great relevance, because of its immediate and future severity, considering the consequent neurological disorders, all of which have multiple causes, including vascular, hemodynamic, inflammatory, and infectious factors. Moreover, these disorders can cause significant neuropsychomotor sequelae and lead to cerebral palsyand or cognitive and behavioral deficits^16^. Transfontanellar ultrasound has become the first-choice modality to study the brain of premature infants and to diagnose PIVH, since it is a cheap, bedside method that does not require patient transport, thus reducing risk of complications^15^.

The aim of this study was to evaluate the association between EOS and increased risk of PIVH in premature patients born at less than or equal to 34 weeks’ gestation.

## METHODS

This retrospective cohort study used data from the medical records of one of the largest hospital groups affiliated to the Brazilian Unified Health System, Grupo Hospitalar Conceição. Since patients born at less than or equal to 34 weeks’ gestation have the highest morbidity and mortality rates^17,18^, the present study collected data of electronic systems from patients born at this gestational age admitted to the neonatal ICU (NICU) of Hospital da Criança Conceição in the city of Porto Alegre, and born from January 2017 to July 2021.

**Figure 1:**
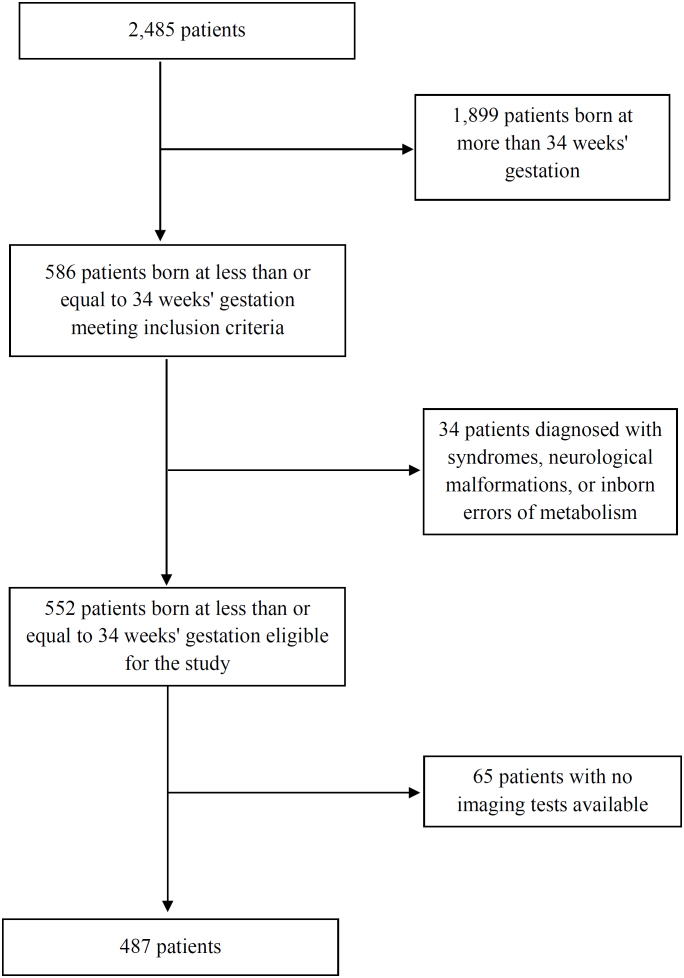
Patients admitted to the neonatal intensive care unit of Hospital da Criança Conceição from January 2017 to July 2021.

Neonatal sepsis may be divided into:

1. EOS, which occurs in the first 24 to 72 hours of life; and
2. LOS, which occurs after the first 72 hours of life. EOS was diagnosed by means of laboratory tests, blood cultures, or diagnosed clinically. It has been a topic of discussion, and the criteria for diagnosis are mostly evaluated together, clinically accompanied by maternal factors and laboratory tests, since the gold standard, blood culture, has a low positive predictive value in neonatology. Microbiological tests are non-specific, and the clinical picture can be similar to several other diseases, such as heart disease. In addition, the maternal history is not always helpful, as it is often not possible to detect infections or changes during pregnancy. For clinical assessment lethargy, feeding intolerance and temperature instability, as well as cardiorespiratory signs, including hypotension and respiratory distress, may be observed^19^. Based on this data, the present study used, for the diagnosis, a collection of blood cultures and supplementary tests, such as leukocyte and platelet count, and PCR. Furthermore, the clinical status was associated with maternal risk factors. Newborns with changes in clinical presentation and laboratory tests and/or germs isolated in blood culture were diagnosed with EOS. If this condition was present, antibiotics were prescribed and initiated immediately. Considering a statistical power of 80%, a significance level of 5%, and a percentage of hemorrhage of 38.6% in patients with one or more episodes of LOS, and 22.8% in patients without LOS, as reported by Poryo et al.^20^, the total sample size was set at 500 patients, of which 84 had one or more episodes of LOS, and 416 had no episodes of LOS. The exclusion criteria were patients with confirmed diagnosis of syndromes, congenital malformations, and inborn errors of metabolism that presented with neurological changes (Figure 1).

The outcome was analyzed based on the results of transfontanellar ultrasounds performed during hospitalization to the NICU. To analyze PIVH, grades of hemorrhage were classified as grades I, II, III and IV, according to the criteria proposed by the Papile et al. classification, based on the location of the hemorrhage and on the presence of ventricular dilation. Grade I refers to hemorrhage restricted to the subependymal germinal matrix. In grade II, the hemorrhage affects the germinal matrix and the ventricles, whereas, in grade III, germinal matrix and ventricular hemorrhages are combined with hydrocephalus. Grade IV, the most severe one, consists of the cases of germinal matrix hemorrhage, ventricular hemorrhage with or without hydrocephalus, and parenchymal hemorrhage^21^. These tests were performed from the first to the fifth day of the newborns’ life, whereas hospitalization time ranged from 1 to 164 days of life.

To analyze the outcome of this study, PIVH was classified as present or absent, not considering its grades. Patients underwent transfontanellar ultrasound according to service routine; therefore, the present study used the first ultrasound after diagnosis of EOS. For the patients not diagnosed with this condition, we used their first transfontanellar ultrasound. During analysis, 65 patients were excluded from the study because they did not have a documented imaging test.

The variables analyzed included both maternal characteristics and those of the mother/child dyad in the pre-, peri-, and postnatal periods. The maternal factors included in the study were: maternal age (in years), maternal education (years of schooling), use of tobacco or drugs (yes or no), number of pregnancies (number of pregnancies), access to prenatal care (yes or no), and number of prenatal visits (number of visits). The following prenatal factors were analyzed: mode of delivery (vaginal or cesarean section), presence of urinary tract infection (yes or no), presence of chorioamnionitis (yes or no), presence of diabetes during pregnancy, including pre gestational and gestational diabetes (yes or no), hypertension (yes or no), premature labor (yes or no), abruption placentae (yes or no), and use of antenatal corticosteroids (yes or no).

The perinatal factors analyzed were sex, gestational age (in days), birth weight (g), Apgar score (from 0 to 10), and intrauterine growth restriction (yes or no). The postnatal variables included: diagnosis of EOS (yes or no), need for mechanical ventilation (yes or no), hyaline membrane disease (yes or no), use of surfactant (yes or no), diagnosis of enterocolitis or meningitis (yes or no), presence of seizures (yes or no), presence of leukomalacia (yes or no), diagnosis of bronchopulmonary dysplasia (yes or no), and hospitalization time (days).

Data were tabulated in the SPSS 2021 software. Quantitative variables were described using position and dispersion measures, whereas qualitative variables were described using frequencies and percentages. Chi-square, Student’s t, and Mann-Whitney tests were used to compare premature infants with and without hemorrhage. Cox models were used to evaluate the occurrence of PIVH considering time from birth to diagnosis of this condition. The patients who did not present this event were censored, and time up to their discharge was calculated. For univariate analysis, the significance level was set at 10%, whereas the multivariate adjusted model considered a significance level of 5%.

The present study was approved by the Research Ethics Committee of Grupo Hospitalar Conceição (CAAE 50041921.5.0000.5530).

## RESULTS

After patients with missing data on the outcome were excluded, a total of 487 patients were assessed. The prevalence of PIVH in the sample was 34.7%. Mean maternal age was similar in children with and without hemorrhage (Table 1). PIVH was found in 60% of newborns whose mothers who did not receive antenatal follow-up.

**Table 1:** Description of maternal and prenatal characteristics of a sample of premature patients born from January 2017 to July 2021.

Variables		Overall n (%)/m (±SD)	Without hemorrhage n (%)/m (±SD)	With hemorrhage n (%)/m (±SD)	p-value
Age					
	In years	27.8 (±6.7)	28.6 (±6.6)	26.99 (±7.0)	0.01*
Schooling					
	In years of study	9.6 (±2.5)	9.8 (±2.5)	9.5 (±2.3)	0.23*
Use of drugs					
	Yes	27 (5.6)	15 (55.6)	12 (44.4)	0.33†
	No	455 (94.4)	300 (65.9)	155 (34.1)	
Smoking					
	Yes	86 (18.0)	60 (69.8)	26 (30.2)	0.33†
	No	392 (82.0)	252 (64.3)	140 (35.7)	
Number of pregnancies					
	In number of pregnancies	2.3 (±1.6)	2.4 (±1.7)	2.1 (±1.4)	0.08*
Prenatal follow-up					
	Yes	457 (93.8)	306 (67.0)	151 (33.0)	0.003†
	No	30 (6.2)	12 (40.0)	18 (60.0)	
Prenatal visits					
	Number of visits	6 (IQR 4–7.7)	6 (IQR 5–8)	5 (IQR 3–7)	<0.001‡
Use of antenatal corticosteroids					
	≥1 dose	351 (72.4)	242 (68.9)	109 (31.1)	0.007†
	No	134 (27.6)	75 (56.0)	59 (44.0)	
Chorioamnionitis					
	Yes	41 (8.4)	19 (46.3)	22 (53.7)	0.01†
	No	446 (91.6)	299 (67.0)	147 (33.0)	
Diabetes during pregnancy					
	Yes	103 (21.1)	72 (69.9)	31 (30.1)	0.27†
	No	384 (78.9)	246 (64.1)	138 (35.9)	
Previous/gestational hypertension					
	Yes	192 (39.4)	129 (67.2)	63 (32.8)	0.48†
	No	295 (60.6)	189 (64.1)	106 (35.9)	
Premature labor					
	Yes	190 (39.0)	120 (63.2)	70 (36.8)	0.42†
	No	297 (61.0)	198 (66.7)	99 (33.3)	
Abruption placentae					
	Yes	59 (12.1)	36 (61.0)	23 (39.0)	0.46†
	No	428 (87.9)	282 (68.9)	146 (34.1)	

* Student’s t test

† Chi-square test

‡ Mann-Whitney test.

IQR: interquartile range (first quartile-third quartile).

Among patients who made use of at least one dose of corticosteroids before birth, 68.9% did not have PIVH (p=0.007). With regard to the mode of delivery, there was no association between this variable and the outcome (p = 0.26). In relation to the diagnosis of chorioamnionitis, hemorrhage was present in 53.7% of newborns whose mothers presented chorioamnionitis (p=0.01) (Table 1).

Concerning the results for perinatal variables, 56.8% of patients with fifth-minute Apgar score below 7 had PIVH (p<0.001) (Table 2). With regard to the association between birth weight and occurrence of PIVH, 54.4% of patients with birth weight below 1,000 g presented the outcome (p<0.001). An analysis of the association between gestational age and occurrence of hemorrhage showed that 57% of patients born at less than 28 weeks’ gestation had PIVH (p<0.001). When it comes to postnatal variables, 41.5% of patients diagnosed with sepsis presented hemorrhage (p<0.001). Among the patients who needed to use surfactants due to the diagnosis of hyaline membrane disease, 50.2% had PIVH (p<0.001). With regard to the presence of leukomalacia, the most common sequel of PIVH, 90.6% of patients diagnosed with this sequela presented PIVH (p<0.001).

**Table 2:** Description of peri- and postnatal characteristics of a sample of premature patients born from January 2017 to July 2021.

Variables		Overall n (%)/m (±SD)	Without hemorrhage n (%)/m (±SD)	With hemorrhage n (%)/m (±DP)	p-value
**Perinatal variables**					
Sex					
	Female	250 (51.3)	168 (67.2)	82 (32.8)	0.36*
	Male	237 (48.7)	150 (63.3)	87 (36.7)	
Mode of delivery					
	Vaginal	118 (24.2)	72 (61.0)	46 (39.0)	0.26*
	Cesarean section	369 (75.8)	246 (66.7)	123 (33.3)	
Category of first minute Apgar score					
	Below 7	267 (54.8)	150 (56.2)	117 (43.8)	<0.001*
	7 or more	220 (45.2)	168 (76.4)	52 (23.6)	
Category of fifth minute Apgar score					
	Below 7	95 (19.5)	41 (43.2)	54 (56.8)	<0.001*
	7 or more	392 (80.5)	277 (70.7)	115 (29.3)	
Birth weight category					
	≤1,000 g	134 (27.5)	61 (45.5)	73 (54.5)	<0.001*
	>1,000 g and <1,500 g	253 (52.0)	170 (67.2)	83 (32.8)	
	≥1,500 g	100 (20.5)	87 (87.0)	13 (13.0)	
Gestational age category					
	<28 weeks	135 (27.7)	58 (43.0)	77 (57.0)	<0.001*
	≥28 and <32 weeks	236 (48.4)	166 (70.3)	70 (29.7)	
	≥32 and 34 weeks	116 (23.8)	94 (81.0)	22 (19.0)	
Intrauterine growth restriction					
	Yes	126 (25.9)	87 (69.0)	39 (31.0)	0.30*
	No	361 (74.1)	231 (64.0)	130 (36.0)	
**Complications during hospitalization**					
Early-onset sepsis					
	Yes	330 (67.8)	193 (58.5)	137 (41.5)	<0.001*
	No	157 (32.2)	125 (79.6)	32 (20.4)	
Mechanical ventilation					
	Yes	245 (50.3)	123 (50.2)	122 (49.8)	<0.001*
	No	242 (49.7)	195 (80.6)	47 (19.45)	
Use of surfactants					
	Yes	221 (45.4)	110 (49.8)	111 (50.2)	<0.001*
	No	266 (54.6)	208 (78.2)	58 (21.8)	
Hyaline membrane disease					
	Yes	229 (47.0)	117 (51.1)	112 (48.9)	<0.001*
	No	258 (53.0)	201 (77.9)	57 (22.1)	
Leukomalacia					
	Yes	32 (6.7)	3 (9.4)	29 (90.6)	<0.001*
	No	449 (93.3)	310 (69.0)	139 (31.0)	
Seizures					
	Yes	123 (25.3)	42 (34.1)	81 (65.9)	<0.001*
	No	364 (74.7)	276 (75.8)	88 (24.2)	
Meningitis					
	Yes	29 (6.0)	17 (58.6)	12 (41.1)	0.43*
	No	458 (94.0)	301 (65.7)	157 (34.3)	
Enterocolitis					
	Yes	65 (13.3)	35 (53.8)	30 (46.2)	0.03*
	No	422 (86.7)	283 (67.1)	139 (32.9)	
Bronchopulmonary dysplasia					
	Yes	119 (24.4)	53 (44.5)	66 (55.5)	<0.001*
	No	368 (75.6)	265 (72.0)	103 (28.0)	
Hospitalization time					
	Days	36 (IQR 21-58)	37 (IQR 24.7-55)	50 (IQR 29-70)	<0.001†

* Chi-square test

† Mann- Whitney test.

IQR: interquartile range (first quartile-third quartile).

Table 3 shows the results of the multivariate analysis for the association between sepsis and PIVH. The variables included in the model were: diagnosis of EOS, gestational age, use of surfactants, and use of antenatal corticosteroids. The presence of EOS leads to a 52% increased risk (95% confidence interval [95%CI] 1.01–2.27) for the occurrence of PIVH, as well as use of surfactants, which increased the risk of this outcome in 72% (95%CI 1.18–2.51). Regarding the variable gestational age, the higher the age, the lower the risk of the outcome, with a hazard ratio of 0.97 (95%CI 0.96–0.98), and, in relation to the use of at least one antenatal corticosteroid dose, reduction in the risk of PIVH remained in 37% (95%CI 0.45–0.86).

**Table 3:** Univariate and multivariate Cox regression model for risk of peri-intraventricular hemorrhage in premature infants born from January 2017 to July 2021.

Variables	Univariate		Multivariate	
	Hazard ratio (confidence interval)	p-value	Hazard ratio (confidence interval)	p-value
Maternal age	0.97 (0.95–0.99)	0.017		
Maternal education	0.96 (0.90–1.03)	0.30		
Use of drugs	1.40 (0.78–2.53)	0.27		
Prenatal follow-up	2.14 (1.30–3.5)	0.002		
First-minute Apgar score	0.87 (0.79–0.90)	<0.001		
Fifth-minute Apgar score’	0.76 (0.70–0.82)	<0.001		
Birth weight	0.99 (0.99–0.99)	<0.001		
Gestational age in days	0.96 (0.96–0.97)	<0.001	0.97 (0.96–0.98)	<0.001
Chorioamnionitis	1.94 (1.24–3.04)	0.004		
Early-onset sepsis	2.24 (1.52–3.29)	<0.001	1.52 (1.01–2.27)	0.04
Use of surfactants	2.84 (2.06–3.90)	<0.001	1.72 (1.18–2.51)	<0.005
Mode of delivery	1.38 (0.98–1.94)	0.58		
Use of antenatal corticosteroids	0.62 (0.45–0.86)	0.004	0.63 (0.45–0.86)	0.004

## DISCUSSION

The results suggest that EOS in children born at less than or equal to 34 weeks’ gestation was an independent predictor of PIVH. According to Gotardo et al.,^22^ the, main findings show a significant increase in the risk of the outcome PIVH, which eventually worsens neuropsychomotor prognosis. Exposure of the premature brain to inflammatory mediators during infectious episodes, such as sepsis and enterocolitis, is related to cerebral hemorrhage, white matter lesions and changes in neurological development, including lower intelligence quotient and cerebral palsy^22,23^. Most of these studies evaluated LOS, an important difference with regard to this study, since an association was found between PIVH and diagnosis of EOS. Studies conducted in the last 10 years revealed that the prevalence of PIVH in the premature population ranged from 20 to 40%, a percentage similar to that observed in the present study. However, these figures are known to be decreasing with the improvement in premature patients’ care^24^.

The present study also found an association between the use of surfactants in patients diagnosed with hyaline membrane disease and the increased occurrence of PIVH. Patients born at lower gestational ages and closer to extreme prematurity belong to the group requiring the use of surfactants, either a single or multiple doses. Therefore, premature infants born at less than 29 weeks’ gestation have increased mortality and morbidity rates, including severe brain injuries, such as PIVH^25^. A recent study identified an increased incidence of peri-intraventricular hemorrhage in newborns with hyaline membrane disease, which would be related to the need for mechanical ventilation and the use of surfactant^26^.

Gestational age as a risk factor for the occurrence or severity of hemorrhage seems to have a logical relationship, as the closer to full-term birth, the lower the risk of developing the outcome. A study published in 2018 showed that prematurity alone, when compared to term birth, increase the risk of PIVH^23^. Furthermore, this condition may be aggravated by low birth weight, low gestational age, and the need for mechanical ventilation^27^. Risk and severity of neonatal cerebral hemorrhage are inversely related to gestational age and birth weight: the incidence of this event is 1% in babies born at 38–43 weeks’ gestation, and 50% in those born at 24–30 weeks’ gestation^28^.

With regard to the reduced risk of hemorrhage in premature infants who received at least one dose of antenatal corticosteroids, this finding corroborates other results already described in the literature. A reduction in the risk, severity, and occurrence of hemorrhage, especially in premature infants born from 28 to 32 weeks’ gestation, has already been associated with the use of antenatal corticosteroids^23,29^.

The results of this study were not sufficient to show an association between chorioamnionitis and increased risk of PIVH. The results found in the univariate analysis demonstrated that chorioamnionitis increases the risk of peri-interventricular hemorrhage; however, after the multivariate analysis, this variable lost statistical significance. Studies demonstrate that maternal ovular infection increases the occurrence of early neonatal sepsis, analyzing the results found that this risk could indeed be related to the increased occurrence of peri-interventricular hemorrhage^30^. Nevertheless, the literature has already found a close relationship between diagnosis of EOS in premature infants and presence of maternal ovary infection^23^. Studies conducted in developing countries found results applicable to pregnancies delivered between 23 and 34 weeks’ gestation, suggesting that gestational age and an inflammatory intrauterine environment play key roles in the occurrence of PIVH and the development of sepsis^24^. Future prospective studies should be carried out to confirm this association. Based on the results presented herein, the occurrence of PIVH does seem to be influenced by the mode of delivery. However, some studies associated cesarean section with reduced risk of PIVH^29^, whereas most studies discuss these conflicting results and the need for further follow-up studies^30,31^.

Due to the retrospective cohort design of the study, no transfontanellar ultrasound scans were obtained for the diagnosis of hemorrhage immediately after the diagnosis of EOS. Transfontanellar ultrasounds were performed according to service routine, aiming to determine the presence or absence of PIVH. Hemorrhage was the outcome studied and, despite acknowledging the importance of classifying this condition into severity grades, it was not possible to do it. Therefore, in this study the outcome was analyzed as presence or absence of PIVH. Prospective and multicenter studies should be conducted to confirm these associations.

The results of this study showed an association between presence of EOSs in preterm infants born before or equal to 34 weeks’ gestation and increased risk of PIVH in this population. Furthermore, a decrease was observed in the occurrence of PIVH among patients who received at least one dose of corticosteroids.
